# Knowledge sharing: nurse managers’ practices

**DOI:** 10.1590/0034-7167-2023-0287

**Published:** 2024-11-22

**Authors:** Ana Lúcia Arcanjo Oliveira Cordeiro, Rosana Maria de Oliveira Silva, Josicélia Dumêt Fernandes, Gilberto Tadeu Reis da Silva

**Affiliations:** I Universidade Federal da Bahia. Salvador, Bahia, Brazil

**Keywords:** Health Information Management, Knowledge Management, Health Services Administration, Nursing Service, Hospital, Nursing Staff, Hospital, Gestión en Salud, Difusión de la Información, Gestión del Conocimiento, Gestión de la Práctica Profesional, Servicio de Enfermería en Hospital

## Abstract

**Objective:**

To analyze how management practices for sharing knowledge are developed in public hospitals in the context of nursing.

**Methods:**

Qualitative research, carried out with 15 nurse managers from six public hospitals, from July to September 2022. Data were collected through semi-structured interviews and analyzed according to content analysis and in a model for sharing knowledge at work.

**Results:**

Knowledge sharing in nursing occurs through intraand extra-organizational training (training, courses and events), use of digital communication tools (media and social networks) and individual and collective contact between professionals during the service (experiences, exchange of experiences and assessment feedback).

**Final considerations:**

Sharing knowledge is relevant to nursing work. To improve it, it must be understood that effective sharing occurs from person to person, in their daily practices, and must be conducted as a strategically planned process by nurse managers.

## RESUMEN

**Objetivo:**

Analizar cómo se desarrollan prácticas de gestión para compartir conocimientos en hospitales públicos en el contexto de la enfermería.

**Métodos:**

Investigación cualitativa, realizada con 15 gerentes de enfermería de seis hospitales públicos, de julio a septiembre de 2022. Los datos fueron recolectados mediante entrevistas semiestructuradas y analizados según análisis de contenido y en un modelo de intercambio de conocimientos en el trabajo.

**Resultados:**

El intercambio de conocimientos en enfermería ocurre a través de capacitación intra y extra organizacional (capacitaciones, cursos y eventos), uso de herramientas de comunicación digital (medios de comunicación y redes sociales) y contacto individual y colectivo entre profesionales durante el servicio (experiencias, intercambio de experiencias y retroalimentación de evaluación).

**Consideraciones finales:**

Compartir conocimientos es relevante para el trabajo de enfermería. Para mejorarlo, es necesario comprender que el compartir efectivo ocurre de persona a persona, en sus prácticas cotidianas, y debe ser realizado como un proceso estratégicamente planificado por los enfermeros gestores.

## INTRODUCTION

Knowledge sharing represents a tool in the organizational knowledge management process, highlighted in the 21^st^ century, to achieve competitive advantages in the market. Therefore, it can be adopted by companies in different production and service segments, including hospital service providers. This management tool involves a web of knowledge of a technical, scientific, philosophical and political nature as well as individual and collective actions, which occur intentionally and collaboratively, in order to create and apply knowledge through the use of various operational means^(1)^.

It is noteworthy that knowledge sharing is transfer of experience, skills and information already acquired and applied in daily service practices, which results in innovation. An organization that encourages knowledge sharing tends to increase innovation capabilities in products, services, processes, businesses and organizational models. Such sharing also favors decision-making and allows expanding knowledge, learning and improving experiences. It also works as a mechanism to convert tacit knowledge into explicit, essential for achieving innovation^(2)^, improving relationships with customers and suppliers, in addition to providing cost reduction in the organization^(3)^.

The adoption of knowledge sharing in organizations has caused significant effects, revealed in several studies^(4-6)^. According to research carried out in China, the environment where knowledge sharing occurs significantly favors a company’s technological innovation capacity^(4)^. In Indonesia, a study carried out with 150 industry entrepreneurs proved that the relationship between knowledge sharing, absorptive capacity, innovation, professional skills and the company’s quality performance was positive and significant in relation to absorptive capacity and professional skill, such that the authors recommended that entrepreneurs improve quality performance through greater knowledge sharing^(5)^. In another research^(6)^, it was found that managers needed to understand the importance of sharing knowledge, since, by promoting interaction between people, technologies and techniques, it is possible to carry out difficult and innovative tasks, with the possibility of triggering positive organizational results, with superior performance assessment and more effective teams, in addition to expanding and strengthening the body of organizational knowledge^(6)^.

Under this understanding, it is observed, within the scope of hospital service provision management, how challenging it has been to share knowledge effectively and constantly, using the tools available in the current context, due to structure complexity, operational processes, culture, organizational climate and structural diversity of hospital organizations. These factors, in many cases, still maintain a rigid, obsolete management model with still incipient levels of information technology.

Nurses inserted in this organizational context face the challenge, from a managerial point of view, of leading the team in an integrated way to apply, produce and share strategic knowledge, given the complexity of care practice, in an environment that is often unfavorable to this development. However, it is possible to observe this professional’s commitment and dedication to implement the culture of knowledge sharing in the service, involving a multidisciplinary team working in the units under their responsibilities, although there is still no concrete systematization and robust organizational support, especially in public hospitals. Research with 414 nurse managers analyzed the relationship between nurse leaders and nurses’ knowledge sharing behavior and recommended strengthening leadership to encourage this behavior in Chinese university hospitals^(6)^.

It is noteworthy that evidence-based practice has been prioritized in nursing work, and it is important to apply scientific knowledge at all times when care is provided and in administration of services in care units. It is also important, at the same time, to strengthen nurses’ autonomy, which, based on updated scientific knowledge, underpins the decision-making process and qualified practices^(7)^, supporting strategic decisions, achievement of service objectives and the adoption of care methodologies. It is understood, therefore, that there are different ways and resources to share knowledge in organizations and in the nursing service, through formal strategies and extra-organizational relationships, both at the individual level, from professional to professional, and at the organizational level, from the individual to the collective.

In view of these considerations, the need for this study is understood in order to contribute to developing strategic actions to encourage and strengthen knowledge sharing. This research, it is worth highlighting, becomes especially relevant at this historical moment of full expansion and appreciation of knowledge in health, sharpened by the pandemic period of countless uncertainties in which potentialities, weaknesses, shortcomings and needs were highlighted, which required construction and reconstruction of practices and highlighted the need for strategic actions to share knowledge.

Furthermore, there is a gap in knowledge disseminated on this topic, with a lack of research carried out in the field, especially in Brazilian hospital organizations. Therefore, it is expected that this study will contribute to filling this gap by expanding reflections on nursing management practice in order to improve integration, increase service quality indicators and the resulting organizational competitiveness. The aim is also to promote reflections on the understanding that each professional is capable of improving both the process and the results of their work, by incorporating, into their practice, learning obtained from the most diverse experiences of their personal life.

The present study was based on Tonet and Paz Model of Knowledge Sharing at Work guidelines^(8)^, which proposes guidance for managers to share knowledge in the context where they operate in an orderly manner, following four phases: initiation, implementation, support and incorporation. The initiation phase is represented by actions to identify and analyze knowledge needs and existing conditions for applying new knowledge and identifying its sources. Implementation is one that promotes integration between sources and recipients of knowledge. In the support phase, opportunities are created to experiment with shared knowledge. In the incorporation phase, the aim is for knowledge to circulate freely among those professionals who will apply it^(8)^. In this model, the clarity with which it exposes situations that may occur and interfere with the sharing process of both personal and organizational origin stands out, involving the source, the recipient, the knowledge itself and the context in which it can be shared.

## OBJECTIVE

To analyze how management practices for sharing knowledge are developed in public hospitals in the context of nursing.

## METHODS

### Ethical aspects

The study was approved under the opinion of the Research Ethics Committee of the *Universidade Federal da Bahia* School of Nursing in December 2021, with its own funding. This study complied with guidelines and standards that regulate research involving human beings, as set out in Resolution 466/2012 of the Brazilian National Health Council and Circular Letter 2/2021/CONEP/SECNS/MoH, Brasília, published on February 24, 2021, which guides procedures in research at any stage in a virtual environment.

Each participant, after hearing the necessary clarifications about the study, agreed to sign the Informed Consent Form in two copies, one being given to the interviewed nurse and the other kept with the researcher. To preserve managers’ identity, the alphanumeric system G for manager was used, followed by the number, according to the order in which interviews were carried out, from G1 to G15.

### Study design and setting

This is a qualitative study, with an exploratory and descriptive design, which analyzes the reports of nurse managers working in public hospitals in a city in northeastern Brazil. The writing of this study followed COREQ^(9)^ checklist guidelines for qualitative studies, which is composed of items divided into three domains: research team and reflexivity; study concept; and analysis and results. After sending requests for consent for the research to 20 medium and large hospitals, six public hospitals agreed to participate in the research.

### Participants and eligibility criteria

The study had 15 nurses as participants, who met the inclusion criteria and agreed to participate. Initially, all nurses were selected, working in the administration of open and closed nursing care units, such as Intensive Care Unit, Emergency Unit and Clinical Unit, Surgical Unit and SMC. Based on the list of personal contacts made available by hospitals’ general nursing management and after interviews had begun, new participants were sought, according to the research’s analytical needs, following the information exhaustion criterion to complete the search for participants. Thus, as a criterion for inclusion of managers, it was considered that they had worked for at least six months in the role, understanding that, after this period in the role, they were already integrated into the organization and more confident in the management process in terms of conducting team and institutional policies. Managers on vacation or on special or gestational leave during the data collection period and those who postponed the interview after three appointments were excluded.

### Data collection and organization

Data collection took place from July to September 2022. After identifying and locating managers, contacts were made to introduce the researchers and the research project, and invitations were sent via email and social networks, whose content explained the research intention, objectives and importance of participating in the study.

The interview script consisted of two parts, the first with sociodemographic information and the second with the following question: describe how your management practices are developed for sharing knowledge in the nursing service. The consent form was signed by participants before interviews began. The interviews, carried out individually, lasted an average of 40 minutes, in a quiet, exclusive and uninterrupted room at the location of the interviewees’ choice and also virtually using Google Meet. They were recorded with participants’ authorization, with the help of a voice recorder on the cell phones of the doctoral and master’s student researchers who carried out the interviews in order to record all the information provided. At the end of the interview, participants had the opportunity to listen to the recordings to authorize transcription or not.

### Data analysis

The data analyzed according to content analysis^(10)^ followed three stages. Firstly, the content of interviews was skimmed to constitute the *corpus*. In the second stage, exhaustiveness, determined by the use of all the content of interviews, representativeness, to express experiences, and interview homogeneity criteria, in search of similarities and relevance of the content to the research objectives, were followed. Subsequently, there was a selection, decomposition and assignment of thematic registration units which, due to similarity, were grouped into three categories and five subcategories. They are: formal means of intraand extra-organizational training (courses and in-service training, courses and scientific events promoted by training institutions and professional associations); digital communication tools (media, social networks and visual communications); and individual and collective contact between professionals during the service (experiences and exchange of experiences between professionals on site) and moment of assessment feedback to organize information and analysis.

The data saturation point was reached when information began to be repeated without new elements being identified in each analysis category. The third stage was carried out through interpretation of results and analysis synthesis. Analysis of results was based on the Tonet and Paz Model of Knowledge Sharing at Work^(8)^, for whom knowledge sharing, more than its transfer from one person to another in an organization, constitutes a process composed of four different phases: initiation, implementation, support and incorporation. It is noteworthy that, at each stage, difficulties permeate, and the content of the knowledge to be transferred, the transmitter and receiver of knowledge and the context in which sharing occurs must be considered.

## RESULTS

A total of 14 women and one man who held administrative positions in nursing care units were interviewed. The length in these positions ranged from 1 to 11 years and that of nursing graduates ranged from 12 to 37 years. They were all experts. These individual characteristics of interviewees facilitated understanding their reports, considering the context to which they were linked, their professional maturity, their experiences and qualifications for the position.

It was identified that, to disseminate knowledge in the nursing service, managers develop managerial actions of practices that favor and encourage professionals to share knowledge in their daily work.

### Formal means of intraand extra-organizational training

#### Courses and events promoted by training institutions and professional associations

The interviewees mentioned the use and promotion of conditions for their teams to participate in courses and scientific events offered in different modalities, with greater emphasis on those offered by the hospital organization in which they worked. They also mentioned specialization courses offered by training institutions and scientific events promoted by professional entities.

*I even started an MBA course in management at the Universidade Católica* [...] *because the need exists even to maintain knowledge, right? So, I’m going to search the networks. I see that COREN always launches a lot of course stuff. I see the SUS itself, right? The SUS university...* [...] *whenever I receive this information, I transfer it to the group. We now have two trained nurses specializing in robotics, because I saw a card, I shared it. In fact, three of them are doing graduate studies, one is already working with robotics.* (G13)

*The main source of information data are technical publications, conferences, events. It’s the most effective way to share as a team. It means doing good planning and involving the team itself, because the experience we have there is that, especially when we involve the technical part, there is a greater interest.* (G15)

[...] *the team even participates by presenting their work. But as scientific days and conferences are held within the hospital, the group also produces this knowledge so that it can be presented to other areas, both in production and sharing, right? This way internally.* (G14)

#### Courses and in-service training

Courses and in-service training promoted by hospitals stood out in interviewees’ reports as continuing education practices to train professionals.

*We hold weekly meetings and other meetings, such as conversation circles, lectures and training. We have meetings with managers who, in each service, present their activities. And we have that media, which is television, which stays in the sectors showing the topics of interest of the moment.* (G1)

*We have also given lectures during shift changes.... we have also done workshops, recreational activities and discussions with continuing education. It is also for people in all areas of the hospital, not only involving nursing, but also physiotherapy, maintenance and hygiene staff. Everyone participates.* (G4)

*Look... with the team, we take classes, we have sessions, right? Case discussion, classes, sessions, there are also recorded educational videos.* (G6)

[...] *we presented, at an event on patient safety in 2021, a work on the hospital experience* [...] *this production of knowledge managed to go beyond, let’s say, the walls of the hospital, we managed to have a positive impact on what we achieved through this publication,* [...] *taking our experience to other hospital units.* (G7)

*I really like working on themes throughout the year for the management development of my team. So, from this perspective, we choose some themes that we would like to develop during the year, and, from then on,* [...] *these themes are covered every fortnight or when there is an interesting theme that goes beyond the scope that I already I spoke.* (G9)

### Digital communication tools

#### Media, social networks and visual communications

According to reports, the communication process using information technology is part of the resources for knowledge interaction and sharing and allows distances to be reduced, in addition to speeding up information and knowledge dissemination in the nursing work environment.

*We also used media. During COVID, we needed to use a lot of videos that were produced. We have panels spread across the hospital, murals with the indicators that are launched.* (G1)

*We put notices on the wall, these innovations too, we make posters at the entrance to the cafeteria, because that’s cool.* (G4)

[...] *we have a WhatsApp group, because now, with the internet and the advancement of technology, it also helps when it comes to getting information out more quickly.* [...] *there is also feedback from the team, which brings me situations in which I may be adjusting difficulties.* (G12)

[...] *generally, we also share the indicators we produce in message groups. The presentations of the indicators that we make for the board are generally also shared with the group so that they are aware of what was produced by them and I highlight what was Surgical production, what was an improvement that we managed to reduce cost consumption.* (G13)

### Individual and collective contact between professionals during the service

#### Experiences and exchange of experiences between professionals on site

Various forms of exchanging experiences and experiences were highlighted by managers in relation to lessons shared with and between co-workers and leaders, in daily work, both spontaneously and programmed.

[...] *using coordination between management areas in a practical way, sharing with the entire care team and the organization as a whole... starting with meetings, conversation circles and other activities... this knowledge is shared among peers.* (G3)

*So, we really have to have good timing, always be up to date, always looking for the most relevant and current things, right? So that we can safely replicate this for the team.* (G1)

[...] *we have a leadership meeting that also aims to develop these leaders.* [...] *so, there we bring new routines, new protocols and their own management development themes.* (G9)

*So, it’s my experience, I try to talk to some co-workers... with more experienced people, with nurses and get guidance, advice. There’s a board that supports us, we go to school, so it’s a way of applying, on a daily basis, here in the moments when I’m guiding the team to enter the surgical center and give some guidance... it’s the way I seek to acquire and in the exchanges that are important, what one has experienced in a place and “Wow, this is cool here, this is interesting”.* (G12)

*So, experience in other places is also a way of acquiring... recently the hospital, we are finalizing the project with another hospital* [...] *in Rio Grande do Sul and a macro project in Brazil, all institutions, working towards patient safety goals.* (G12)

*And the professionals who exchange their own doubts, their own information who share their experiences, who inspire other units to reproduce those successful experiences.* (G15)

[...] *we go there formally, as I told you in the meetings, because as we are training, we are also multiplying it for other co-workers, right?* (G8)

#### Moment of assessment feedback

During the work process supervision and assessment, managers reported on-site knowledge sharing:

[...] *we accurately assess all infection control protocols. So, it’s also an opportunity for improvement because we train there. We discover that, sometimes, the team does not yet have some type of knowledge, there is also an opportunity for us to have a chat with the employee.* (G2)

[...] *when we carry out clinical audits, tracers and practice supervision, we also multiply this and in the moments when we think about meeting institutional needs*. (G8)

*There are some difficulties and we assess here what is best, what is the strategy, in many moments, a guideline is given, but it can be revised and we adapt, seeing if it worked, there was a difficulty like that.* (G12)

## DISCUSSION

In the scope of nursing work, it was evident in this study that courses and events promoted by training institutions and professional associations, and those offered by hospitals, including training, were identified as opportunities to acquire and share knowledge. However, both the organization and the professional need to address needs and acquire up-to-date knowledge to remain qualified and ensure employability. However, according to this study’s theoretical foundations, there is no guarantee of socialization of the knowledge acquired individually to other co-workers, nor of its application in the service without a context conducive to building cooperative relationships and mutual trust, leadership and formal support given to sharing by the organization^(8)^.

It is known that, during courses, knowledge is shared between participants and instructors, but, at the organizational level, what is expected is that the knowledge acquired individually is shared with other service professionals and applied. In the age of easily accessible information, it is still necessary to share knowledge among professionals to carry out work effectively and apply and produce knowledge, since tacit knowledge belongs individually to people^(11)^.

It was observed that managers considered the courses and lectures as sharing practices, supporting what has been evidenced: the lack of consensus on how knowledge is shared between people, as is the case with the interpretation of the expressions “knowledge sharing” and “information sharing”, treated as synonyms. The first, it is worth highlighting, requires planned action that enables the process of granting knowledge to be used and that is needed by another person, whereas the second demands significant structures capable of generating knowledge in people or in the organization^(8)^.

This study considers that, in managers’ reports, the first phase of the sharing process called initiation permeates the entire knowledge sharing process in the nursing service. At this stage, knowledge needs and existing opportunities to employ different or new knowledge are identified, as well as to locate sources where this knowledge can be found, such as experiences, courses and lectures mentioned, which are offered by the organization, or are of interest to professionals. In the model by Tonet and Paz^(8)^, the initiation phase appears to be more relevant, as it depends on the degree of difficulty in locating demands, perceiving opportunities to transfer useful knowledge already mastered, but still restricted to people or work units as well as in unfavorable situations for the organization, such as losses, repeated errors and insufficient performance.

It becomes challenging for organizations and managers to optimize and reuse knowledge that is often restricted to the individual sphere of professionals or limited to some sectors, and must be shared to increase the efficiency, effectiveness and resolution of the services provided.

In this regard, this professional training process precedes the managerial role to promote conditions to enable knowledge sharing in the service. It is worth highlighting, however, that this comes from actions to identify possible individual and organizational barriers to sharing, as well as the need to apply new knowledge and sources where it can be found, including short and long-term courses, congresses, lectures, seminars, studies, among others.

It is important to highlight the study by Huie, Cassaberry and Rivera^(11)^, which confirms the need for managers to understand knowledge sharing in order to be inductors and encourage this practice. According to the study, this understanding allows expanding the level of organizational knowledge without forgetting, however, that the more an organization knows, the more it can learn.

In this study, it is considered that interviewees implemented sharing when they mentioned promoting conditions for their teams to participate in courses and scientific events, use of digital communication resources and exchange of experiences between professionals, where links were established between the source of knowledge and the recipient. According to Tonet and Paz foundations^(8)^, in the implementation phase, one must seek to improve interpersonal, intergroup and organizational communication processes, create a climate conducive to voluntary involvement, cooperation and the establishment of trust between people and the organization, and encourage the acquisition of new skills, among others.

The digital communication tools used by interviewees, such as media, social networks and visual communications, were highlighted as accessible and practical resources, whose use was intensified during the pandemic, to share knowledge. A study shows that, in the health field, as in other areas, information and communication technologies have been contributing and positively transforming work processes in the Brazilian Health System (*Sistema Único de Saúde*), as they facilitate learning and multiplier teaching. They are also responsible for positive implications in the process of continuing education of healthcare professionals with easy access to teaching and learning^(12)^.

Other technologies facilitate knowledge application and sharing, such as artificial intelligence in healthcare, already available today to support professionals with a variety of tasks, from administrative workflow to clinical documentation and patient care. Such technologies also provide specialized support, such as image analysis, automation of medical devices and patient monitoring, and can be combined with advances in wireless technology and smartphones^(13)^.

Research carried out in auditing and consulting companies in the Middle East confirmed hypotheses that information technologies and social networks favor the knowledge management cycle, such as acquisition, creation, storage, sharing and application in companies. Furthermore, it has been proven that they have an additional impact on organizational performance, with benefits also for society, emphasizing the open culture of sharing^(14)^.

Currently, the growth in the use of virtual environments in learning has been evident with advances in mobile technologies that access the Internet, such as cell phones and tablets, to obtain information, rationalize and make the most of time in the educational process. It is a practice increasingly used among healthcare professionals to interact, produce, access and disseminate information^(15)^. França, Rabello and Magnago^(15)^ suggest expanding the proposal to adopt these technologies for current use as strategic tools for managing education and work in health.

It is important to highlight WhatsApp^®^, an application, cited by interviewees as a means of sharing knowledge. A study^(16)^ considered that this free application is superior to other applications and platforms, since multimedia information involves recipients of encrypted messages with secrecy and security, making it possible to promote health and share important information about health and disease, quickly and at low cost. Thus, knowledge between healthcare professionals and between them and patients can be effectively shared through this means^(16)^. A network society, it is worth pointing out, is built by relationships that arise from affinities, interests and common ideas, with man as the center because he is this social being. In scientific communication, sharing is capable of building and reconstructing concepts^(17)^.

From managers’ reports, the practice of sharing knowledge during the service among professionals emerged individually and in collective moments, such as meetings, conversation circles, experiences, advice, guidance from experts and in moments of feedback on assessments by leaders. The exchange of knowledge and experiences between professionals in organizations is important for integration between individuals and for improving services. The role of nurse managers was observed as a source of knowledge to be shared with professionals on their team, after the individual performance assessment process.

According to the interpretation of authors Tonet and Paz^(8)^, who guide this discussion, sharing knowledge in organizations is a main process that occurs in work groups, in the service space where practice takes place. In this way, a professional conveys what they know to the people they work with so that they receive, assimilate and share this knowledge.

Tonet and Paz^(8)^ propose actions for planning to be followed for implementation: identification and dimension of knowledge to be shared; strategies; control of variables related to the source and receiver; mastery of skills considered requirements; belief in the viability and opportunity of sharing; beware of recurrence of known problems; and criteria for assessing the progress made.

Managers’ reports mentioned the time for reviewing protocols and feedback on assessments from team professionals, during which knowledge needs and opportunities to review and rectify those that have been passed on and that still deserve adjustments and corrections are identified. Therefore, this moment was understood in this study as the support phase, characterized by actions that promote the application of shared knowledge for guidance until the assimilation and development of necessary skills^(8)^. It is noteworthy that the shared knowledge incorporation phase, although not made explicit in interviewees’ reports, is implicit in approaches to use knowledge in professional nursing practice, as recipients apply what they learned in the daily routine of their work. In this conception, the organizational context must have environments conducive to free circulation of knowledge so that dialogue is favorable and frequent and that each person is sensitive to speaking and listening, acting as a source and receiver of knowledge^(8)^.

At the organizational level, a study^(18)^ highlighted that the practice of mentoring knowledge sharing and experience improvement, in addition to training participants to perform their functions and contributing to improving interpersonal relationships. This practice between a more experienced professional (mentor) and a less experienced professional (disciple, mentee, *protégé*) contributes to developing the service or a specific activity.

In interviewees’ reports, all working in public hospitals, similarities were observed regarding managerial actions to promote knowledge sharing, with emphasis on courses and participation in events promoted by training institutions and professional associations and courses and training in services where managers themselves were the source and multipliers of knowledge. In this study, we sought to understand, based on the foundations of organizational knowledge management, that knowledge sharing is a process with several phases to be properly planned, coordinated, applied and systematized to improve professionals’ performance, promote innovation and quality of nursing care in the hospital.

### Study limitations

As a limitation of this study, interviews with nurses only from public hospitals are considered, considering that the private network’s experience could add additional elements to the practice of sharing knowledge at work.

### Contributions to nursing, health or public policies

The experiences presented can be thought-provoking and serve as references to other realities. The discussions in this study highlight possibilities for managerial actions by nurses who need to develop a strategic plan in order to promote, in the nursing service, environment capable of consolidating information and knowledge sharing for the creation and application of new knowledge that flows easily in the development of work practices. This study explains possibilities for applying the knowledge sharing process in healthcare organizations, especially in hospital settings, and contributes to filling the gap in the production of this knowledge in the area.

## FINAL CONSIDERATIONS

Managers used management practices to share knowledge in hospital nursing services, with the understanding that this occurs during formal and programmed actions, through actions that involve the team collective, through intraand extra-organizational training such as courses, events, training, digital communication tools, media, social networks and visual communications. Furthermore, such sharing, to be effective, depends on individual and collective contact between professionals during the service, through experiences and exchange of experiences and in assessment feedback.

However, for these practices to be able to improve service performance, it is necessary for managers to understand knowledge sharing as a phased process that requires planning, coordination, application and systematization, and must be conducted and applied from person to person in daily work. It is also necessary to consider important elements for operationalization, such as professionals’ profile, skills and knowledge domain, source, recipients’ profile regarding the needs, skills and level of experience, complexity and dimension of the knowledge to be shared, and whether the environmental conditions are, in fact, favorable to the actions. It is noted that most managers highlighted actions that seek professional thematic updates, which are essential, but which may not reach the level of transformation intended in the service, especially if the acquisition of new knowledge has not been strategically planned, shared and applied to meet the service’s needs, as recommended by knowledge management in organizations.

It is expected that this study will contribute to the understanding and reflection of nurse managers, in order to improve professionals’ performance, promote innovation and quality of nursing care, through managing the knowledge sharing applied in hospital organizations.
