# Supplementary material for: Knowledge sharing: nurse managers’ practices
Source: Rev Bras Enferm. 2024 Nov 22;77(5):e20230287. doi: 10.1590/0034-7167-2023-0287 (PMC11653875; doi:10.1590/0034-7167-2023-0287)
Supplement: Translation

# Translation: Compartilhamento do conhecimento: práticas de gestores de enfermagem

**DOI:** 10.1590/0034-7167-2023-0287pt

**Authors:** Ana Lúcia Arcanjo Oliveira Cordeiro, Rosana Maria de Oliveira Silva, Josicélia Dumêt Fernandes, Gilberto Tadeu Reis da Silva

**Affiliations:** I Universidade Federal da Bahia. Salvador, Bahia, Brasil

**Keywords:** Gestão em Saúde, Compartilhamento de Conhecimento, Gestão do Conhecimento, Gestão da Prática Profissional, Serviço Hospitalar de Enfermagem

## Abstract

**Objetivo:**

Analisar como são desenvolvidas, em hospitais públicos, práticas de gestão para o compartilhamento do conhecimento no contexto da enfermagem.

**Métodos:**

Pesquisa qualitativa, realizada com 15 enfermeiros gestores de seis hospitais públicos, de julho a setembro 2022. Os dados foram coletados através de entrevistas semiestruturadas e analisados segundo a análise de conteúdo e em um modelo para compartilhamento do conhecimento no trabalho.

**Resultados:**

O compartilhamento do conhecimento na enfermagem ocorre mediante capacitação intra e extraorganizacional (treinamentos, cursos e eventos), uso de ferramentas de comunicação digital (mídias e redes sociais) e contato individual e coletivo entre profissionais durante o serviço (vivências, troca de experiências e feedback de avaliações).

**Considerações finais:**

O compartilhamento do conhecimento é relevante para a atuação da enfermagem. Para aperfeiçoá-lo, deve-se apreender que o compartilhamento efetivo ocorre de pessoa a pessoa, no cotidiano de suas práticas, devendo ser conduzido como um processo estrategicamente planejado pelos enfermeiros gestores.

## INTRODUÇÃO

O compartilhamento de conhecimento representa uma ferramenta do processo de gestão do conhecimento organizacional, em destaque no século XXI, para o alcance de vantagens competitivas no mercado. Dessa forma, pode ser adotado por empresas de diversos segmentos de produção e serviços, inclusive por prestadoras de serviços hospitalares. Essa ferramenta de gestão envolve uma teia de conhecimentos de natureza técnica, cientifica, filosófica e política, bem como ações individuais e coletivas, que ocorrem de forma intencional e colaborativa, a fim de criar e aplicar o conhecimento mediante a utilização de diversos meios operacionais^(1)^.

Destaca-se que o compartilhamento de conhecimento é a transferência de experiência, habilidades e informações já adquiridas e aplicadas nas práticas cotidianas dos serviços, que resultam em inovação. Uma organização que incentiva o compartilhamento de conhecimento tende a aumentar as capacidades de inovação nos produtos, serviços, processos, negócios e nos modelos organizacionais. Tal compartilhamento favorece ainda a tomada de decisões e permite ampliar conhecimentos, saberes e aprimorar experiências. Funciona, também, como mecanismo para converter o conhecimento tácito em explícito, imprescindível para o alcance da inovação^(2)^, melhoria do relacionamento com clientes e fornecedores, além de proporcionar a redução de custos na organização^(3)^.

A adoção do compartilhamento de conhecimento nas organizações causou efeitos significativos, revelados em diversos estudos^(4-6)^. Segundo pesquisa desenvolvida na China, o ambiente onde o compartilhamento de conhecimento ocorre favorece, de forma relevante, a capacidade de inovação tecnológica de uma empresa^(4)^. Na Indonésia, estudo desenvolvido com 150 empresários da indústria comprovou que a relação entre compartilhamento de conhecimento, capacidade de absorção, de inovação, habilidade profissional e desempenho de qualidade da empresa foi positiva e significativa em relação à capacidade de absorção e habilidade profissional, de tal modo que os autores recomendaram aos empresários melhoria do desempenho de qualidade por meio de maior compartilhamento de conhecimento^(5)^. Em outra pesquisa^(6)^, constatou-se a necessidade do gestor compreender a importância do compartilhamento de conhecimento, visto que, ao promover a interação entre pessoas, tecnologias e técnicas, ele possibilita a realização de tarefas difíceis e inovadoras, com possibilidade de desencadear resultados organizacionais positivos, com avaliação de desempenho superior e equipes mais eficazes, além de ampliar e fortalecer o corpo de conhecimento organizacional^(6)^.

Sob esse entendimento, observa-se, no âmbito da gestão de prestação de serviço hospitalar, o quanto tem sido desafiador compartilhar conhecimentos de forma efetiva e constante, mediante a utilização das ferramentas disponíveis no contexto atual, devido à complexidade da estrutura, dos processos operacionais, da cultura, do clima organizacional e da diversidade estrutural das organizações hospitalares. Esses fatores, em muitos casos, ainda mantêm um modelo de gestão rígido, obsoleto e com níveis de tecnologia da informação ainda incipientes.

O enfermeiro inserido neste contexto organizacional vive o desafio, do ponto de vista gerencial, de conduzir a equipe de forma integrada para aplicar, produzir e compartilhar conhecimentos estratégicos, diante da complexidade da prática assistencial, em ambiente, muitas vezes, desfavorável a este desenvolvimento. Contudo, é possível observar empenho e dedicação desse profissional para implantar a cultura do compartilhamento de conhecimentos no serviço, envolvendo equipe multiprofissional atuante nas unidades, sob suas responsabilidades, embora ainda não exista sistematização concreta e um robusto apoio organizacional, principalmente em hospitais públicos. Pesquisa com 414 enfermeiros gestores analisou a relação entre enfermeiros-líderes e o comportamento de compartilhamento de conhecimento de enfermeiros e recomendou o fortalecimento de lideranças para estimular essa conduta em hospitais universitários chineses^(6)^.

Destaca-se que a prática baseada em evidência tem sido priorizada no trabalho da enfermagem, sendo importante a aplicação do conhecimento científico em todos os momentos em que são realizados os cuidados e nas ações de administração dos serviços das unidades assistenciais. Importante também, paralelamente, fortalecer a autonomia do enfermeiro, que, baseado em conhecimento científico atualizado, fundamenta o processo de tomada de decisão e das práticas qualificadas^(7)^, subsidiando decisões estratégicas, alcance de objetivos no serviço e a adoção de metodologias para o cuidado. Compreende-se, destarte, que existem diversas formas e recursos para compartilhar conhecimentos nas organizações e no serviço de enfermagem, através de estratégias formais e de relações extraorganizacionais, tanto no âmbito individual, de profissional para profissional, quanto do individual para o coletivo, na esfera organizacional.

Frente a essas considerações, entende-se a necessidade do presente estudo a fim de contribuir com o desenvolvimento de ações estratégicas para estímulo e fortalecimento do compartilhamento de conhecimento. Esta pesquisa, vale ressaltar, torna-se especialmente relevante neste momento histórico de plena expansão e valorização do conhecimento na saúde, aguçada pelo período pandêmico de inúmeras incertezas em que foram evidenciadas potencialidades, fragilidades, carências e necessidades, o que demandou a construção e reconstrução de práticas e sublinhou a necessidade de ações estratégicas para o compartilhamento dos conhecimentos.

Ademais, observa-se uma lacuna em relação ao conhecimento divulgado acerca da presente temática, com carência em pesquisas desenvolvidas em campo, especialmente em organizações hospitalares brasileiras. Assim, espera-se que este estudo contribua para o preenchimento dessa lacuna ao ampliar reflexões sobre a prática de gestor de enfermagem, a fim de melhorar a integração, elevar os indicadores de qualidade do serviço e a consequente competitividade organizacional. Almeja-se, também, promover reflexões sobre o entendimento de que cada profissional é capaz de aprimorar tanto o processo quanto os resultados do seu trabalho, ao incorporar, na sua prática, as aprendizagens obtidas nas mais diversas experiências de sua vida pessoal.

O presente estudo foi fundamentado nas diretrizes do Modelo do Compartilhamento do Conhecimento no Trabalho de Tonet e Paz^(8)^, que propõe a orientação de gestores para o compartilhamento do conhecimento no contexto onde atuam de forma ordenada, seguindo quatro fases: *iniciação, implementação, apoio* e *incorporação*. A fase de *iniciação* é representada por ações para a identificação e análise das necessidades de conhecimentos e condições existentes para aplicação de novos conhecimentos e identificação de suas fontes. A *implementação* é aquela em que se promove a integração entre as fontes e destinatários do conhecimento. Na fase de *apoio*, são criadas oportunidades de experimentação do conhecimento compartilhado e, na fase de *incorporação,* almeja-se que o conhecimento possa circular livremente entre aqueles profissionais que irão aplicá-lo^(8)^. Destaca-se, neste modelo, a clareza com que expõe situações passíveis de ocorrer e interferir no processo de compartilhamento tanto de origem pessoal quanto organizacional, envolvendo a fonte, o destinatário, o próprio conhecimento e o contexto em que este pode ser compartilhado.

## OBJETIVO

Analisar como são desenvolvidas, em hospitais públicos, práticas de gestão para o compartilhamento de conhecimento no contexto da enfermagem.

## MÉTODOS

### Aspectos éticos

O estudo foi aprovado sob parecer do Comitê de Ética em Pesquisa da Escola de Enfermagem da Universidade Federal da Bahia em dezembro de 2021, com financiamento próprio. Atendeu às diretrizes e normas que regulamentam as pesquisas envolvendo seres humanos, preceituadas pela Resolução 466/2012 do Conselho Nacional de Saúde e pelo Ofício Circular Nº 2/2021/CONEP/SECNS/MS, Brasília, publicado em 24 de fevereiro de 2021, que orienta procedimentos em pesquisas com qualquer etapa em ambiente virtual.

Cada participante, após ouvir os esclarecimentos necessários sobre o estudo, concordou em assinar o Termo de Consentimento Livre e Esclarecido em duas vias, sendo uma entregue ao enfermeiro entrevistado e a outra mantida sob posse da pesquisadora. Para preservar a identidade dos gestores, utilizou-se o sistema alfanumérico G de gestor, seguido do número, conforme ordem de realização das entrevistas, de G1 a G15.

### Tipo de estudo e cenário

Trata-se de um estudo qualitativo, com delineamento exploratório e descritivo, que analisa o relato de gestores de enfermagem atuantes em hospitais públicos de um município do Nordeste brasileiro. A redação deste estudo seguiu as diretrizes do *checklist* COREQ^(9)^ para estudos qualitativos, o qual é composto por itens divididos em três domínios: equipe de pesquisa e reflexividade; conceito do estudo; e análise e resultados. Após envio das solicitações de anuência para a pesquisa a 20 hospitais de médio e grande porte, seis hospitais públicos concordaram em participar da pesquisa.

### Participantes e critérios de elegibilidade

O estudo teve como participantes 15 enfermeiros, que atenderam aos critérios de inclusão e que aceitaram participar. Inicialmente, foram selecionados todos os enfermeiros, em exercício laboral, em cargos de administração de unidades abertas e fechadas de assistência de enfermagem como: Unidade de Terapia Intensiva, Unidade de Emergência e Internação Clínica, Cirúrgica e CME. A partir da relação de contatos pessoais disponibilizados pela gerência geral de enfermagem dos hospitais e após iniciadas as entrevistas, buscou-se novos participantes, conforme necessidade analítica da pesquisa seguindo o critério de exaustão das informações, para a finalização de busca dos participantes. Assim, considerou-se, como critério para inclusão dos gestores, a atuação por, no mínimo, 6 meses na função, por entender que, após esse período no cargo, já estavam integrados na organização e mais seguros no processo de gestão quanto à condução da equipe e às políticas institucionais. Foram excluídos gestores em férias ou de licença especial ou gestacional no período da coleta de dados e aqueles que postergaram a entrevista após três agendamentos.

### Coleta e organização dos dados

A coleta de dados ocorreu de julho a setembro 2022. Após identificação e localização dos gestores, foram realizados contatos para a apresentação das pesquisadoras e do projeto de pesquisa e encaminhados os convites através de *e-mail* e redes sociais, cujo conteúdo explicava a intenção da pesquisa, os objetivos e a importância da participação no estudo.

O roteiro da entrevista constou de duas partes, sendo a primeira com informações sociodemográficas e a segunda com a seguinte questão: descreva como são desenvolvidas as suas práticas de gestão para o compartilhamento do conhecimento no serviço de enfermagem. O termo de consentimento foi assinado pelos participantes antes do início das entrevistas. As entrevistas, realizadas individualmente, duraram em média 40 minutos, em sala silenciosa, exclusiva e sem interrupções no local de escolha dos entrevistados e, também, virtualmente utilizando a plataforma *Google Meet.* Elas foram gravadas com autorização dos participantes, com auxílio do gravador de voz dos celulares das pesquisadoras doutoranda e mestrandas que realizaram as entrevistas, a fim de registrar todas as informações fornecidas. Ao término da entrevista, os participantes tiveram a oportunidade de ouvir as gravações, para autorizar ou não a transcrição.

### Análise dos dados

Os dados analisados de acordo com a Análise de Conteúdo^(10)^ seguiram três etapas. Na primeira, realizou-se a leitura flutuante do conteúdo das entrevistas, para obter a constituição do *corpus*. Na segunda etapa, foram obedecidos os critérios de exaustividade, determinada pelo uso de todo o conteúdo das entrevistas, de representatividade, para que expressasse as experiências, e de homogeneidade das entrevistas, em busca das similaridades e pertinência do conteúdo aos objetivos da pesquisa. Em seguida, houve recorte, decomposição e atribuição das unidades de registro temático, que, pela similaridade, foram agrupadas em três categorias e cinco subcategorias. São elas: meios formais de capacitação intra e extraorganizacional (cursos e treinamentos em serviço, cursos e eventos científicos promovidos por instituições formadoras e entidades de classe; ferramentas de comunicação digital (mídias, redes sociais e comunicações visuais); e contato individual e coletivo entre profissionais durante o serviço (vivências e troca de experiências entre profissionais in loco) e momento do *feedback* de avaliações, para organização das informações e análise.

Alcançou-se o ponto de saturação dos dados quando as informações começaram a se repetir sem que novos elementos fossem identificados em cada categoria de análise. A terceira etapa foi efetivada mediante interpretação dos resultados e síntese da análise. A análise dos resultados fundamentou-se no Modelo do Compartilhamento do Conhecimento no Trabalho de Tonet e Paz^(8)^, para os quais o compartilhamento do conhecimento, mais do que seu repasse de uma pessoa para outra em uma organização, constitui um processo composto por quatro diferentes fases: iniciação, implementação, apoio e incorporação. Ressalta-se que em cada fase permeia dificuldades, devendo ser considerados o conteúdo do conhecimento a ser transferido, o transmissor e o receptor do conhecimento e o contexto em que ocorre o compartilhamento.

## RESULTADOS

Foram entrevistadas 14 mulheres e um homem que ocupavam cargos de administração de unidades assistenciais de enfermagem. O tempo nos referidos cargos variou de 01 a 11 anos e o de graduado em enfermagem, de 12 a 37 anos. Todos eram especialistas. Essas características individuais dos entrevistados facilitaram o entendimento de seus relatos, considerando o contexto a que estavam vinculados, a maturidade profissional, suas experiências e qualificações para o cargo.

Identificou-se que, para divulgar conhecimentos no serviço de enfermagem, os gestores desenvolvem ações gerenciais de práticas que favorecem e estimulam o profissional a compartilhar conhecimentos no cotidiano do seu trabalho.

### Meios formais de capacitação intra e extraorganizacional

#### Cursos e eventos promovidos por instituições formadoras e entidades de classe

Os entrevistados mencionaram a utilização e promoção de condições de participação de suas equipes em cursos e eventos científicos oferecidos em diversas modalidades, com maior destaque para os ofertados pela organização hospitalar em que atuavam. Referiram, ainda, cursos de especializações oferecidos por instituições formadoras e eventos científicos promovidos por entidades de classe.

*Eu até iniciei um curso de um MBA em gestão lá na Universidade Católica* [...] *porque a necessidade existe até para a manutenção mesmo do conhecimento, não é? Então eu vou buscando mesmo nas redes. Vejo que o COREN sempre lança muita coisa de curso. Vejo o próprio SUS, né? A universidade do SUS...* [...] *Sempre que eu recebo essas informações, transfiro para o grupo. A gente tá agora com duas enfermeiras formadas especialistas em robótica, porque eu vi um card, eu compartilhei. Na verdade, três delas estão fazendo pós, uma já está trabalhando com robótica.* (G13)

*A principal fonte de dados de informações são publicações técnicas, congressos, eventos. É a forma mais eficaz de compartilhar enquanto equipe. É fazendo um bom planejamento e envolvendo a própria equipe, porque a vivência que a gente tem lá é que: principalmente quando a gente envolve a parte técnica, existe um interesse maior.* (G15)

[...] *a equipe participa, inclusive, apresentando os trabalhos. Mas, como dentro do hospital são feitas jornadas científicas, congressos, o grupo também produz esses conhecimentos para que sejam apresentados para as outras áreas, tanto na produção como no compartilhamento, né? Dessa forma internamente.* (G14)

#### Cursos e treinamentos em serviço

Cursos e treinamentos em serviço promovidos pelos hospitais se destacaram nos relatos dos entrevistados enquanto práticas de Educação Continuada para capacitar profissionais.

*A gente faz encontros semanais e os demais encontros, como rodas de conversa, palestras e instrumentaliza. Nós temos reuniões com os gestores que, em cada serviço, apresentam suas atividades. E nós temos aquela mídia que é a televisão que fica nos setores passando os temas de interesse do momento.* (G1)

*Temos feito palestras utilizando também na hora da passagem de plantão.... Temos feito também oficinas, atividades lúdicas e discute com educação continuada. É também para as pessoas de todas as áreas do hospital, não somente envolvendo a enfermagem, como o pessoal da fisioterapia, manutenção, higienização. Todos participam.* (G4)

*Olha... com a equipe, a gente faz as aulas, tem sessões né? Discussão de caso, aulas, sessões, tem vídeos gravados, educativos também.* (G6)

[...] *apresentamos, em um evento sobre segurança do paciente no ano de 2021, um trabalho sobre a experiência do hospital* [...] *essa produção do conhecimento conseguiu extrapolar, digamos, os muros do hospital, conseguimos ter um impacto positivo nisso que a gente conseguiu através dessa publicação,* [...] *levando essa nossa experiência para outras unidades hospitalares.* (G7)

*Eu gosto muito de trabalhar temáticas no ano para o desenvolvimento gerencial da minha equipe. Então, nessa perspectiva, a gente escolhe alguns temas que a gente gostaria de desenvolver no ano, e, a partir daí,* [...] *essas temáticas elas são abordadas quinzenalmente, ou quando tem algum tema também interessante que foge desse escopo que eu já falei*. (G9)

### Ferramentas de comunicação digital

Mídias, redes sociais e comunicações visuais

Segundo os relatos, o processo de comunicação com a utilização de tecnologia da informação faz parte dos recursos para interação e compartilhamento de conhecimento e permite reduzir distâncias, além de agilizar a disseminação de informações e conhecimentos no ambiente de trabalho da enfermagem.

*A gente usou também mídias; na época da COVID, a gente teve necessidade de utilizar bastante vídeos que foram produzidos. Nós temos painéis espalhados no hospital, os murais com os indicadores que são lançados.* (G1)

*A gente coloca no mural os avisos, essas inovações também, faz cartazes na entrada do refeitório, porque isso é legal.* (G4)

[...] *a gente tem um grupo de WhatsApp, porque agora, com a internet e o avanço das tecnologias, ajuda também na hora de disparar uma informação mais rápida.* [...] *Tem um feedback também da equipe, que traz para mim situações em que eu possa estar ajustando dificuldades.* (G12)

[...] *geralmente compartilha também nos grupos de mensagem os indicadores que a gente produz. As apresentações dos indicadores que a gente faz para a diretoria são geralmente compartilhadas também com o grupo, para que eles fiquem cientes do que foi produzido por eles e coloco em destaque o que foi de produção Cirúrgica, o que foi de melhoria que a gente conseguiu reduzir o consumo de custo.* (G13)

### Contato individual e coletivo entre profissionais durante o serviço

#### Vivências e troca de experiências entre profissionais in loco

Várias formas de troca de experiências e vivências foram apontadas pelos gestores em relação aos ensinamentos compartilhados com e entre colegas e líderes, no cotidiano do trabalho, tanto de forma espontânea como programada.

[...] *utilizando a articulação entre as áreas da gerência de forma prática, compartilhando com toda a equipe assistencial e da organização como um todo... partindo da realização de reuniões, rodas de conversa entre outras atividades... É feito o compartilhamento desse conhecimento entre os pares.* (G3)

*Então a gente tem que ter realmente um timing, estar sempre antenado, estar sempre buscando as coisas mais relevantes e mais atuais né? Para que a gente possa, de forma segura, replicar isso para equipe.* (G1)

[...] *nós temos uma reunião de liderança que também tem como objetivo o desenvolvimento desses líderes.* [...] *Então ali a gente traz rotinas novas, protocolos novos e as temáticas de desenvolvimento gerencial deles mesmo.* (G9)

*Então é a minha vivência, eu procuro conversar com alguns colegas ...com pessoas mais experientes, com enfermeiros e ter orientações, aconselhamento. Tem a diretoria que apoia, a gente vai para os estudos, então, é, a forma de estar aplicando, é, no dia a dia, aqui nos momentos em que eu estou orientando a equipe entrar no centro cirúrgico e dar algumas orientações ... É a forma que eu busco adquirir e nas trocas que são importantes, o que um vivenciou em um lugar e: Poxa, isso aqui é legal, isso aqui é interessante.* (G12)

*Então, a vivência também em outros lugares é uma forma de adquirir... recentemente o hospital, a gente está finalizando o projeto com um outro hospital* [...] *do Rio Grande do Sul e um projeto macro no Brasil, todas as instituições, trabalhando as metas de segurança do paciente.* (G12)

*E os profissionais que trocam suas próprias dúvidas, as suas próprias informações que compartilham as experiências, que vão inspirando outras unidades a reproduzirem aquelas experiências que foram de sucesso.* (G15)

[...] *a gente aí vai formalmente, como eu te falei nas reuniões, porque à medida que a gente tá capacitando, a gente também tá multiplicando pra outros colegas né?* (G8)

#### Momento do feedback de avaliações

Durante a supervisão e avaliação do processo de trabalho, os gestores relataram o compartilhamento de conhecimentos *in loco:*

[...] *a gente avalia exatamente todos os protocolos do controle de infecção. Então também é uma oportunidade de melhoria porque ali a gente treina, a gente descobre que, às vezes, a equipe não detém ainda algum tipo de conhecimento, tem dúvida também uma oportunidade de a gente fazer um bate bola com o colaborador.* (G2)

[...] *no momento em que a gente faz as auditorias clínicas os tracers* [refere-se a rastreadores] *e a supervisão da prática a gente também multiplica isso. E nos momentos em que a gente pensa em atender às necessidades institucionais.* (G8)

*De algumas dificuldades e a gente vai avaliando aqui o que é melhor, qual a estratégia, em muitos momentos, uma orientação é passada, mas ela pode ser revista e a gente vai adaptando, olhando se deu certo, teve uma dificuldade assim*. (G12)

## DISCUSSÃO

No âmbito do trabalho da enfermagem, ficou evidenciado, neste estudo, que os cursos e eventos promovidos por instituições formadoras e entidades de classe, e os oferecidos pelos hospitais, incluindo os treinamentos, foram apontados como oportunidades de adquirir e compartilhar conhecimentos. Observa-se, contudo, tanto para a organização como para o profissional, a necessidade de suprir carências e adquirir conhecimentos atualizados para se manter qualificado e assegurar a empregabilidade. No entanto, conforme os fundamentos teóricos deste estudo, não existe garantia de socialização do conhecimento adquirido individualmente, para os demais colegas da equipe, tampouco de sua aplicação no serviço, sem um contexto propício à construção de relacionamentos cooperativos e de confiança mútua, liderança e o apoio formal dado ao compartilhamento pela organização^(8)^.

Sabe-se que, durante os cursos, ocorrem compartilhamentos de conhecimentos entre os participantes e com os instrutores, mas, no âmbito organizacional, o que se espera é que os conhecimentos adquiridos individualmente sejam compartilhados com os demais profissionais do serviço e aplicados. Na era da informação de fácil acesso, ainda é necessário compartilhar o conhecimento entre os profissionais para realizar o trabalho de forma eficaz e aplicar e produzir conhecimentos, visto que o saber tácito pertence individualmente às pessoas^(11)^.

Observou-se que os gestores consideraram os cursos e palestras como práticas de compartilhamento, corroborando o que vem sendo evidenciado: a inexistência de consenso sobre como ocorre o compartilhamento do conhecimento entre as pessoas, assim como acontece com a interpretação das expressões *compartilhamento de conhecimento* e *compartilhamento de informação*, tratadas como sinônimos. A primeira, vale ressaltar, exige ação planejada que viabilize o processo de concessão do conhecimento para ser utilizado e que seja necessário a outra pessoa, enquanto a segunda demanda estruturas significantes capazes de gerar conhecimentos nas pessoas ou na organização^(8)^.

Este estudo considera que, nos relatos dos gestores, a primeira fase do processo de compartilhamento denominada *iniciação* permeia todo o processo de compartilhamento do conhecimento no serviço de enfermagem. Nesta fase, identificam-se necessidades de conhecimentos e as oportunidades existentes para empregar saberes diferentes ou novos, assim como para localizar fontes onde esses conhecimentos poderão ser encontrados, a exemplo das vivências, cursos e palestras citados, que são oferecidos pela organização, ou de interesse do profissional. No modelo de Tonet e Paz^(8)^, apresenta-se como mais relevante a fase de *iniciação,* na medida que depende do grau de dificuldade existente de localizar demandas, perceber oportunidades de transferir os conhecimentos úteis já dominados, mas ainda restritos a pessoas ou unidades de trabalho, assim como, em situações desfavoráveis para a organização, como prejuízos, erros repetidos e desempenhos insuficientes.

Torna-se desafiador para as organizações e gestores otimizar e reutilizar conhecimentos que, não raro, estão restritos à esfera individual dos profissionais ou limitados a alguns setores, devendo ser compartilhados para ampliar a eficiência, eficácia e resolutividade dos serviços prestados.

Nesse sentido, antecede este processo de capacitação profissional o papel gerencial para promover condições de viabilizar o compartilhamento de conhecimento no serviço. Vale ressaltar, contudo, que isso advém de ações no sentido de identificar possíveis barreiras individuais e organizacionais para o compartilhamento, bem como necessidades de aplicar novos conhecimentos e fontes onde possam ser encontrados, incluindo os cursos de curta e longa duração, congressos, palestras, seminários, estudos, entre outros.

Importante ressaltar o estudo de Huie, Cassaberry e Rivera^(11)^, o qual confirma a necessidade de gestores compreenderem o compartilhamento de conhecimento para serem indutores e incentivar esta prática. Segundo o estudo, essa compreensão permite expandir o nível de conhecimento organizacional sem esquecer, todavia, de que quanto mais uma organização sabe, mais ela pode aprender.

Considera-se neste estudo que os entrevistados implementaram o compartilhamento quando referiram a promoção de condições para participação de suas equipes em cursos e eventos científicos, uso de recursos de comunicação digital e troca de experiências entre os profissionais, onde foram estabelecidos vínculos entre a fonte detentora do conhecimento e o destinatário. De acordo com os fundamentos de Tonet e Paz ^(8)^, na fase de implementação deve-se buscar: aperfeiçoar os processos de comunicação interpessoal, intergrupal e organizacional; criar clima propício ao envolvimento voluntário, à cooperação e ao estabelecimento da confiança entre as pessoas e organização; e estimular a aquisição de novas competências, entre outras.

As ferramentas de comunicação digital utilizadas pelos entrevistados, como as mídias, redes sociais e comunicações visuais, foram apontadas como recursos acessíveis e práticos, cujo uso foi intensificado durante a pandemia, para compartilhar conhecimentos. Estudo aponta que, no campo da saúde, como em outras áreas, as tecnologias da informação e comunicação vêm contribuindo e transformando de maneira positiva os processos de trabalho no Sistema Único de Saúde brasileiro, por serem facilitadoras de aprendizagem e multiplicadoras do ensino. São responsáveis, além disso, por implicações positivas no processo de educação permanente de profissionais da saúde com facilidade de acesso ao ensino-aprendizagem ^(12)^.

Outras tecnologias facilitam a aplicação e o compartilhamento de conhecimentos, como a inteligência artificial na saúde, já disponível atualmente para apoiar profissionais com uma variedade de tarefas, desde fluxo de trabalho administrativo até documentação clínica e atendimento ao paciente. Tais tecnologias fornecem ainda suporte especializado, como análise de imagens, automação de dispositivos médicos e monitoramento de pacientes, e podem ser aliadas aos avanços da *tecnologia sem fio* e dos *smartphones* ^(13)^.

Pesquisa realizada em empresas de auditoria e consultoria do Oriente Médio confirmou hipóteses de que as tecnologias da informação e as redes sociais favorecem o ciclo de gestão do conhecimento: aquisição, criação, armazenamento, compartilhamento e aplicação nas empresas. Ademais, comprovou-se que exercem impacto adicional no desempenho organizacional, com benefícios também para a sociedade, enfatizando a cultura aberta de compartilhamento ^(14)^.

Atualmente, tem sido evidenciado o crescimento do uso de ambientes virtuais na aprendizagem com os avanços das tecnologias móveis que acessam a Internet, como celulares e *tablets*, para obter informações, racionalizar e aproveitar o tempo no processo educativo. Trata-se de uma prática cada vez mais utilizada entre os profissionais de saúde para interagir, produzir, acessar e disseminar informações ^(15)^. França, Rabello e Magnago ^(15)^ sugerem a ampliação da proposta de adoção dessas tecnologias para uso corrente como ferramentas estratégicas para a gestão da educação e do trabalho na saúde.

Importante destacar o aplicativo *WhatsApp*®, citado pelos entrevistados como meio de compartilhar conhecimento. Estudo ^(16)^ considerou que esse aplicativo gratuito é superior a outros aplicativos e plataformas, visto que as informações multimídia envolvem os receptores de mensagens criptografadas com sigilo e segurança, possibilitando promover a saúde e compartilhar informações importantes sobre saúde-doença, com rapidez e baixo custo. Assim, o conhecimento entre os profissionais da saúde e destes com os pacientes pode ser compartilhado efetivamente por essa via^(16)^. Uma sociedade em rede, vale pontuar, é construída pelas relações que surgem de afinidades, interesses e ideias comuns, tendo o homem como o centro por ser este ser social. Na comunicação científica, o compartilhamento é capaz de construir e reconstruir conceitos^(17)^.

Emergiu, dos relatos dos gestores, a prática de compartilhar conhecimentos durante o serviço entre profissionais de forma individual e em momentos coletivos como: reuniões, rodas de conversas, vivências, aconselhamentos, orientações de experts e nos momentos do *feedback* de avaliações pelas lideranças. A troca de conhecimentos e de experiências entre os profissionais nas organizações é importante para integração entre os indivíduos e para a melhoria dos serviços. Observou-se o papel do enfermeiro gerente como fonte possuidora do conhecimento a ser compartilhado com os profissionais da sua equipe, após o processo de avaliação de desempenho individual.

De acordo com a interpretação das autoras Tonet e Paz ^(8)^, que norteiam esta discussão, o compartilhamento do conhecimento nas organizações é um processo principal, que ocorre nos grupos de trabalho, no espaço do serviço onde se efetiva a prática. Dessa forma, um profissional repassa o que sabe a pessoas com as quais trabalha com o objetivo de que recebam, assimilem e compartilhem este conhecimento.

Tonet e Paz ^(8)^ propõem ações para o planejamento a ser seguido para implementação: identificação e dimensão do conhecimento a ser compartilhado; estratégias; controle de variáveis relacionadas à fonte e ao receptor; domínio de competências consideradas requisitos; crença na viabilidade e oportunidade do compartilhamento; cuidado com a recorrência de problemas conhecidos; e critérios de avaliação do progresso obtido.

Os relatos dos gestores mencionaram o momento de revisão de protocolos e do *feedback* de avaliações dos profissionais da equipe, durante o qual são identificadas necessidades de conhecimentos e de oportunidades para rever e retificar aqueles que foram repassados e que ainda mereçam ajustes e correções. Portanto, tal momento foi entendido neste estudo como a fase de *apoio*, caracterizada por ações que promovem a aplicação do conhecimento compartilhado para orientação, até a assimilação e o desenvolvimento das habilidades necessárias^(8)^. Destaca-se que a fase de *incorporação* do conhecimento compartilhado, embora não explicitada nos relatos dos entrevistados, está implícita nas abordagens sobre o uso do conhecimento na prática profissional da enfermagem, visto que o destinatário aplica o que aprendeu na rotina diária do seu trabalho. Nesta concepção, o contexto organizacional deve dispor de ambientes propícios para uma livre circulação do conhecimento, de modo que o diálogo seja favorável e frequente e que cada um esteja sensível para falar e ouvir, atuando como fonte e receptor de conhecimentos^(8)^.

No âmbito organizacional, estudo^(18)^ ressaltou que a prática do *mentoring* propicia o compartilhamento de saberes e o aprimoramento de experiências, além de capacitar os participantes para o desempenho das suas funções e contribuir para a melhoria nos relacionamentos interpessoais. Esta prática entre um profissional mais experiente (mentor) e outro menos experiente (discípulo, mentorado, *protégé*) contribui para o desenvolvimento do serviço ou de uma atividade específica.

Observou-se, nos relatos dos entrevistados, todos atuantes em hospitais públicos, similaridades sobre as ações gerenciais para promover o compartilhamento do conhecimento, com destaque para realização de cursos e participações em eventos promovidos por instituições formadoras e entidades de classe e os cursos e treinamentos em serviços onde os próprios gestores eram a fonte e os multiplicadores do conhecimento. Buscou-se entender, neste estudo, com base nos fundamentos da gestão do conhecimento organizacional, que o compartilhamento do conhecimento é um processo com várias fases a serem devidamente planejadas, coordenadas, aplicadas e sistematizadas para melhorar o desempenho dos profissionais, promover a inovação e a qualidade da assistência de enfermagem no hospital.

### Limitações do Estudo

Considera-se, como limitação deste estudo, a realização de entrevistas com enfermeiros apenas de hospitais públicos, considerando que a experiência da rede privada poderia agregar elementos adicionais à pratica do compartilhamento do conhecimento no trabalho.

### Contribuições para a Área

As vivências e experiências apresentadas podem ser indutoras de reflexão e servir de referências para outras realidades. As discussões deste estudo evidenciam possibilidades para ações gerenciais de enfermeiros, que precisam elaborar plano estratégico de maneira a fomentar, no serviço de enfermagem, ambiente capaz de consolidar o compartilhamento de informação e conhecimento para a criação e aplicação de novos saberes, que fluam com facilidade no desenvolvimento das práticas laborais. Este estudo explicita possibilidades de aplicação do processo de compartilhamento do conhecimento nas organizações de saúde, especialmente no contexto hospitalar, e contribui para preencher lacuna da produção desse conhecimento na área.

## CONSIDERAÇÕES FINAIS

Os gestores utilizaram práticas de gestão para o compartilhamento do conhecimento nos serviços de enfermagem dos hospitais, com o entendimento que este ocorre durante ações formais e programadas, mediante ações que envolvem o coletivo da equipe, através de capacitação intra e extraorganizacional como cursos, eventos, treinamento, ferramentas de comunicação digital, mídias, redes sociais e comunicações visuais. Ademais, tal compartilhamento, para ser efetivo, depende do contato individual e coletivo entre profissionais durante o serviço, através de vivências e troca de experiências e nos *feedbacks* de avaliações.

Contudo, para que essas práticas sejam capazes de melhorar o desempenho do serviço, é necessário o entendimento, por parte dos gestores, do compartilhamento do conhecimento como um processo de fases que exige planejamento, coordenação, aplicação e sistematização, devendo ser conduzido e aplicado de pessoa a pessoa, no cotidiano do trabalho. É necessário, também, considerar elementos importantes para a operacionalização, tais como: perfil, habilidades e domínio do conhecimento do profissional, fonte, perfil do destinatário referente às necessidades, habilidades e nível de experiência; complexidade e dimensão do conhecimento a ser compartilhado; e se as condições do ambiente são, de fato, favoráveis às ações. Registra-se o fato de a maioria dos gestores ter ressaltado ações que buscam atualizações temáticas profissionais, as quais são imprescindíveis, mas que podem não atingir o nível de transformação pretendida no serviço, principalmente se a aquisição de novos conhecimentos não tenha sido estrategicamente planejada, compartilhada e aplicada para atender às necessidades do serviço, como preconiza a gestão do conhecimento nas organizações.

Espera-se que este estudo contribua para o entendimento e reflexão de enfermeiros gestores, no sentido de melhorar o desempenho dos profissionais, promover a inovação e a qualidade da assistência de enfermagem, através da gestão do compartilhamento de conhecimentos aplicados nas organizações hospitalares.
